# Impact of media literacy education on knowledge and behavioral intention of adolescents in dealing with media messages according to Stages of Change

**Published:** 2015-01

**Authors:** NARJES GERAEE, MOHAMMAD HOSSEIN KAVEH, DAVOD SHOJAEIZADEH, HAMID REZA TABATABAEE

**Affiliations:** 1 Department of health education and promotion, School of Health, Shiraz University of Medical Sciences, Shiraz, Iran;; 2 Department of health education and promotion, School of Health, Tehran University of Medical Sciences, Tehran, Iran;; 3 Department of epidemiology, School of Health, Shiraz University of Medical Sciences, Shiraz, Iran

**Keywords:** Media literacy, Education, Adolescents, knowledge

## Abstract

**Introduction**: Mass media influence the health behaviors of adolescents. Evidence shows that traditional strategies such as censorship or limitation are no longer efficient; therefore, teaching media literacy is the best way to protect adolescents from harmful effects. The aim of this pilot study was to evaluate the effects of a media literacy training program on knowledge and behavioral intention of a sample of female students according to the stages of change in dealing with media messages.

**Methods**: The study was conducted based on a pre-test and post-test control group design. Some 198 female students including 101 in the intervention group and 97 in the control group participated in this study. The educational program was run using interactive teaching-learning techniques. Data collection was performed using a validated and reliable self-administered questionnaire in three phases including a pre-test, post-test, 1 and post-test, 2. The research data was analyzed through SPSS statistical software, version 14 using both descriptive and inferential statistics.

**Results**: The results of the study showed a significant increase (p=0.001) in the intervention group’s knowledge mean scores after the training program. On the other hand, the difference was not significant in the control group (p=0.200). A considerable percentage of the participants, in the intervention and control groups, were in pre contemplation and contemplation stages in the pre-test (64 and 61, respectively). After the intervention, however, a significant improvement (p=0.001) was observed in the intervention group’s stages of change compared to that in the control group. The distribution of the control group students regarding the stages of change was similar to that in the pre-test.

**Conclusion**: The study findings revealed that the planned education programs are efficient to improve the adolescents’ knowledge and behavioral intention in dealing with mass media messages.

## Introduction

The main feature of the 21st century is media-saturated culture and provision and ease of access to different types of media for everybody, particularly children and adolescents (1-4).

In general, mass media are the result of the people’s need to satisfy such requirements as gaining news and information, entertainment, and socialization. However, the media are not the mirror of reality and their content is not always complete, accurate, and unbiased (5, 6).

Nowadays, in addition to consuming the old media, such as TV, children and adolescents also spend a lot of time on new types of media (7, 8). Thus, concerns for the adolescents are increasing in this regard. Some issues related to these concerns include the effect of the media on violence, violent behaviors, and crimes (9, 10), sexual relationships (11), educational performance (12), body image (13), diet, increasing prevalence of obesity and being involved in sedentary activities (14), drug abuse and smoking (15), alcohol abuse (16), food preferences (17), and change in the conversational language structure (5).

Some media authorities believe that such problems can be solved through limitation and censorship. However, censorship and limitation are not desirable responses to the concerns about the mass media and their effects on the children and adolescents (18).

General health specialists have also made use of various strategies for adjusting the effects of the media on health. Up to now, such approaches as regulation of the media contents, limitation of the children’s media consumption, and social marketing, have been utilized to address the problem (7).

Since government laws and regulations and changing the media contents are not appropriate strategies for monitoring media consumption and, at the same time, reduction of the adolescents’ exposure to the media is not always practical, parents feel concerned about their children’s media utilization, because children mostly use the media at home. Therefore, parents’ role, as well as the children’s perception, should be taken into account in directing children’s media consumption (19-21). Nevertheless, studies have shown that in the Iranian society, parents do not highly interfere in their children’s watching TV and using computer games, Internet, and other media (22). Thus, cognitive and motivational backgrounds should be created in the adolescents so that they react toward the media content as well as function spontaneously. One of the important ways to achieve this aim is training the children and adolescents on media literacy.

Many organizations, such as American Academy of Pediatrics, Center for Disease Control and Prevention, Office of National Drug Control Policy, UNESCO, European Commission, and European Parliament and a lot of media organizations, such as Center for Media Literacy (CML), Association for Media Literacy (AML), and National Association for Media Literacy Education (NAMLE), have strong statements regarding media literacy (8, 21).

Media literacy is defined as the “ability to understand, analyze, evaluate, and create media messages”. Media literacy training increases the individuals’ doubt about the media content (23). After all, existence of the individuals with high media literacy leads to increase in the media quality because such individuals require more realistic messages of higher quality (5).

Evidence shows that the interventions which have been based on the theoretical concepts are more effective in comparison to those which have been based on behavior. Moreover, considerable effectiveness of the interventions requires new methodologies and state of the art in order to repeat and develop findings (24).

Up to now, various change theories have been developed, providing frameworks for interventions to help people gain positive healthy behaviors. Overall, individual beliefs and purposes are the main elements of two main theories on changes in health-oriented behaviors: Theory of Planned Behavior (TPB) and Transtheoretical Model (TTM) (25).

Transtheoretical model has been defined as a framework which aims to direct the content and scheduling of the designed interventions for improving and facilitating healthy behaviors (24).

In general, transtheoretical model consists of four constructs: 1- stages of change: temporal readiness for behavior modification, 2- decisional balance: relative importance of perceived advantages and disadvantages of change, 3- situational self-efficacy: trust in the individual capability for behavioral change in positive social and emotional situations, and 4- processes of change: behavioral and experimental strategies people employ in order to improve through the stages (26, 27).

“Stages of change” is the main construct of the transtheoretical model. This model considers behavioral changes as a process rather than a dichotomous phenomenon (28). “Stages of change” is an important construct because it is the representative of time dimension. In the past, behavioral change was considered as a separate event; for instance, quitting smoking, alcohol, or overeating (26). On the other hand, the transtheoretical model assumes change as a dynamic phenomenon which is created over time and due to movement through several distinct stages. Besides, the individuals should make multiple attempts for behavior change in order to achieve complete success (26, 29, 30).

As mentioned above, in the stages of change model, individuals should move through several stages in order to change their behavior. Each stage is a distinct point in the individuals’ readiness for change and it is assumed that they should do a set of activities in a certain period of time in order to move on to the next stage (25).

In spite of the increasing trend of growth of the mass media in Iran, unfortunately no attempts have been made in this regard. Considering this gap, performing educational, as well as research activities, seems to be essential in our country. Therefore, the present study aims to describe the mean score of knowledge and the distribution of behavioral intention of a sample of Iranian female students in exposure to media messages, regarding the stages of change before and after receiving media literacy training. 

## Methods

As media literacy training has not been executed in Iran up to now, this study was conducted as a pilot study, using a randomized, controlled, and educational trial design. The study participants included 198 female students in four state secondary schools in Shiraz, Iran. The students were selected through random sampling. After all, a sample of 198 students was determined for the study with 101 in the intervention and 97 in the control group.

The study data were collected, using a questionnaire which was completed by both groups in three phases including before, immediately and one month after the training program. The content validity of the questionnaire was approved, using the ideas of 5 experts of the field. The questionnaire consisted of two parts: the first including the Knowledge questions with 7 case-based, essay-type, open-ended questions which were calculated using the analytical scoring method. The overall scores ranged from 0 to 5. Twenty percent of questionnaires were randomly selected and the knowledge construct was scored by two independent raters, and 78 percent agreement was achieved between raters. In this study, knowledge implied the ability to remember the information about the effects of media messages and different possible reactions toward them, the techniques utilized in creating the media messages, surface and hidden meanings of the messages and different possible interpretations. The students’ stages of change were assessed by describing a correct situation regarding appropriately and critically dealing with media messages, and posing a question about the conformity of the students’ present behavior to that situation. In this way, the students who mentioned that they had not even thought about it were categorized in the “pre contemplation” group, while those who stated that they had thought about the situation but had not done anything about it were classified in the “contemplation” group. In addition, the students who said that they had talked to the informed individuals and had planned to do something were put in the “preparation” category. Finally, the students who claimed that they had acted properly for almost one month were classified in the “act” category and those who stated that they had acted properly for more than 6 months were categorized in the “maintenance” group.

The training program was conducted in 3 sessions using active teaching/learning methods and the students were also provided with booklets.

Since most of the students in both groups, after the initial analyses, were found to be in the pre contemplation stage, the training strategies were focused on increasing the students’ awareness of the media on the adolescents’ health, different possible reactions toward the media, correct reaction to the media, and the outcomes of having active, critical reactions to the media messages in the students’ present and future lives.

**Statistical Analysis**

The collected data were analyzed through SPSS statistical software, version 14. As K-S Test showed that the distribution of the data was not normal, non-parametric tests such as Mann Whitney and Friedman were used. 

## Results

The students of the two groups were similar regarding age, sex, and kind of school. The pre-test analyses revealed no significant difference between the two groups regarding knowledge score (p=0.2). The mean score of students’ knowledge in the intervention group, being 0.67±0.42 in the pre-test, increased to 3.15±1.13 in the first post-test. Their mean score of knowledge was 2.87±0.89 in the second post-test. The mean score of knowledge in the control group remained relatively the same through the three phases. Using Freidman and Wilcoxon test, we found that the changes in knowledge mean scores within and between the study groups were statistically significant (p=0.001) (Table.1).

**Table 1:** Knowledge mean scores in the groups before and after the training program

Test Groups	Pre-test Mean±SD	Post-test 1 Mean±SD	Post-test 2 Mean±SD
Intervention N=101	0.67±0.42	3.15±1.13	2.87±0.89
Control N=97	0.64±0.56	0.68±0.59	0.66±0.57
p	0.200	0.001	0.001

The results of Chi-square test showed no significant difference (χ^2^=0.78, df=3, p=0.8) between the two groups regarding their frequency distribution according to their intentional behavior status based on the stages of change. Most of the students in both groups were in the pre contemplation stage (64.35% in the intervention and 62.88% in the control group) and a small number of students were in the maintenance stage (2.97% in the intervention and 2.06% in the control group).

After the intervention, 62.37% and 12.87% of the intervention group students were in the preparation and action stages in the first post-test, and 56.43 and 9.90 in the second post-test, respectively. In the control group, on the other hand, the frequency distribution of the students regarding the stages of change in the post-tests were similar to the pre-test and more than half of the students were still in the pre contemplation stage 

The statistical analysis showed significant improvement of the intervention group students regarding the stages of change (p=0.001). However, no significant difference was observed in the frequency distribution of the control group students regarding the stages of change before and after the intervention (Table 2).

**Table 2:** Frequency distribution of the students regarding the stages of change before and after the educational intervention

Groups Stages of change	Pre-test		Post-test1		Post-test2	
	Intervention N=101 F (%)	Control N=97 F (%)	Intervention N=101 F (%)	Control N=97 F (%)	Intervention N=101 F (%)	Control N=97 F (%)
Pre contemplation	65 (64.35)	61 (62.88)	6 (5.94)	63 (64.94)	10 (9.90)	61 (62.88)
Contemplation	15 (14.85)	17 (17.52)	9 (8.91)	15 (15.46)	16 (15.84)	15 (15.46)
Preparation	11 (10.89)	12 (12.37)	63 (62.37)	12 (12.37)	57(56.43)	10 (10.30)
Action	7 (6.93)	5 (5.15)	13 (12.87)	5 (5.15)	10 (9.90)	7 (7.21)
Maintenance	3 (2.97)	2 (2.06)	10 (9.90)	2 (2.06)	8 (7.92)	4 (4.12)
p	*χ^ 2 ^=0.78 0.8		*χ^ 2 ^=115.78 0.001		*χ^ 2 ^=101.26 0.001	

* Statistical test was performed after merging the two last groups (action & maintenance)

## Discussion

The present study investigated the effectiveness of a training program based on the “stages of change” construct of the transtheoretical model, designed in order to improve the knowledge and behavioral intention of the students in exposure to media messages. The study findings revealed the effectiveness of the training program in improving the intervention group students for having an active, critical reaction toward the media messages.

Knowledge is often considered as a prerequisite and predisposing factor to behavioral change. Knowledge is considered as an essential attribute of behavior, and higher rates of knowledge are correlated with higher rates of positive behavior.

Evidence, on the other hand, shows the efficacy of planned educational interventions in knowledge enhancement to facilitate acquiring desired behaviors (31).

The low levels of students’ knowledge about media literacy, as detected in the pre-test phase of this study, imply the lack of related educational programs in our country and students’ need for such essential programs. 

Therefore, the significant increase in knowledge mean score of the intervention group in both phases after the training program is in favor of the efficacy of such programs in improving students’ knowledge about media.

This finding is in line with a large number of studies about media literacy trainings. For instance, Kupersmidt and Scull concluded that even a one-day workshop on media literacy education was effective on the participants’ knowledge of media literacy (2).

According to the study results concerning the stages of change, a considerable percentage of the participants were in precontemplation and contemplation stages regarding having an active reaction in exposure to media messages in the pre-test. After the intervention, however, a significant improvement was observed in the intervention group’s stages of change in comparison to that in the control group.

Kupersmidt and Scull performed a study and showed a significant reduction in the behavioral intention for alcohol and tobacco abuse in the students who had participated in media literacy training program (32).

Furthermore, based on the studies by TQ, Tein et al. (2010) and Kupersmidt et al. (2011) to evaluate the adolescents’ media literacy and its relationship with smoking, alcohol abuse, and their future vulnerability, having media literacy was accompanied by less drug and alcohol abuse (33, 34).

“Stages of change” construct of the transtheoretical model is based on the assumption that training can improve the individuals’ development through the stages. In this model, each stage represents how much the training has been accepted by the individuals and how effective it has been (35). Moreover, the participants should perform appropriate tasks at the right time in order to move on to the next stage. This implies that the individuals need special strategies, called the processes of change, in each stage (36).

According to this model, the individuals in the pre contemplation stage need information about the dangers of their present behavior, while those in the following stages require practical recommendations regarding how to change their behavior (35). Thus, processes such as awareness increasing should be applied in order to help the individuals move from the pre contemplation to the contemplation stage (26).

The findings of the current study showed that using awareness increasing strategies regarding daily media consumption, negative effects of the media, and different possible reactions toward them led to a significant improvement in the intervention group students’ development from pre contemplation to contemplation and preparation stages. 

It has been assumed that moving through the stages is related to the factors associated with each particular behavior. Therefore, identification and measurement of the effective factors in moving through the stages, including motivation for change, self-confidence, self-efficacy, and social support, can also be beneficial in designing more influential interventions (37).

## Conclusion

In conclusion, the low levels of students’ knowledge about media literacy and their distributions at precontemplation and contemplation stages at pre-test, showed the lack of any sufficient educational programs in Iranian schools. This study revealed the adolescents' need for a theory-based Media Literacy education program. 

**Ethical aspects of the study**

As there was no obligation to mention the name on the questionnaires, and students were assured that their responses will be confidential and also the data were analyzed collectively, therefore there was no need to fill the consent form. 

## References

[1] Potter WJ (2008). Media Literacy.

[2] Scull TM, Kupersmidt BJ (2011). An Evaluation of a Media Literacy Program Training Workshop for Late Elementary School Teachers. J Media Lit Educ.

[3] Fuchs M (2008). An Introduction to Media Literacy. J Media Lit Educ.

[4] Wan G, Gut DM (2008). Media Use by Chinese and U.S. Secondary Students: Implications for Media Literacy Education. Theory Into Practice.

[5] Ulaş AH, Epçaçan C, Koçak B (2012). The concept of “Media Literacy” and an evaluation on the necessity of media literacy education in creating awareness towards Turkish language. Procedia-Social and Behavioral Sciences.

[6] Duran RL, Yousmman B, Walsh KM, Walsh KM, Longshore MA (2008). Holistic Media Education: An Assessment of the Effectiveness of a College Course in Media Literacy. Communication Quarterly.

[7] Bergsma LJ, Carney ME (2008). Effectiveness of health-promoting media literacy education: a systematic review. Health Educ J.

[8] Oxstrand B (2009). Sweden Media literacy education- a discussion about media education in western countries, Europe and Sweden.

[9] Huesmann LR, Taylor LD (2006). The role of media violence in violent behavior. Public Health.

[10] Felson RB (1996). Mass media effects on violent behavior. Annual Review of Sociology.

[11] Pinkleton BE, Austin EW, Chen YY, Cohen M (2012). The Role of Media Literacy in Shaping Adolescents’ Understanding of and Responses to Sexual Portrayals in Mass Media. J Health Commun.

[12] Schmidt ME, Vandewater EA (2008). Media and Attention, Cognition, and School Achievement. Future Child.

[13] Yamamiya Y, Cash TF, Melnyk SE, Posavac HD, Posavac SS (2005). Women’s exposure to thin-and beautiful media images: body image effects of media-ideal internalization and impact-reduction interventions. Body Image.

[14] Wharf Higgins J, Begoray D (2012). Exploring the Borderlands between Media and Health: Conceptualizing ‘Critical Media Health Literacy. Journal of Media Literacy Education.

[15] Primack BA, Gold MA, Land SR, Fine MJ (2006). Association of Cigarette Smoking and Media Literacy about Smoking among Adolescents. J Adolesc Health.

[16] Austin EW, Chen MJ, Grube JW (2006). How does alcohol advertising influence underage drinking? The role of desirability, identification and skepticism. J Adolesc Health.

[17] Dovey TM, Taylor L, Stow R, Boylan EJ, Halford JC (2011). Responsiveness to healthy television (TV) food advertisements/commercials is only evident in children under the age of seven with low food neophobia. Appetite.

[18] Heins M, Cho C (2003). Media Literacy: An Alternative to Censorship.

[19] Brown JD, Knight JL (2006). Media literacy has potential to improve adolescents’ health. Adolescent Health.

[20] Mendoza K (2009). Surveying parental mediation: connections, challenges and questions for media literacy. JMLE.

[21] Bier MC, Schmidt SJ, Shields D, Zwarun L, Sherblom S, Primack B (2011). School-based Smoking Prevention with Media Literacy: A Pilot Study. JMLE.

[22] Javadi F, Eghbali B (2007). Parental supervision on children and adolescents’ use of visual media. Quarterly Journal of Communication Research.

[23] Austin EW, Chen YY, Pinkleton BE, Johnson JQ (2006). Benefits and costs of cannel one in a middle school setting and the role of media literacy training. Pediatrics.

[24] Wright AJ, Velicer WF, Prochaska JO (2009). Testing the predictive power of the transtheorical model of behavior change applied to dietary fat intake. Health Educ Res.

[25] Floyd J, Zebrowski PM, Flamme GA (2007). Stages of change and stuttering: a preliminary view. J Fluency Disord.

[26] Glanz K, Rimer BK, Viswanath K (2008). Theory of reasoned action, theory of planned behavior, and the integrated behavior model.

[27] Noia JD, Contento IR, Prochaska JO (2008). Computer-mediated intervention tailored on transtheoretical model stages and processes of change increases fruit and vegetable consumption among urban African-American adolescents. Am J Health Promot.

[28] Molaison EF, Yadrick MK (2003). Stages of change and fluid intake in dialysis patients. Patient Educ Couns.

[29] Kim YH (2004). Korean adolescents’ exercise behavior and its relationship with psychological variables based on stages of change model. J Adolesc Health.

[30] Bernardes S, Caramori PR (2011). Stages of change for fruit and vegetables intake among patients with atherosclerotic disease. Appetite.

[31] Kaveh MH, Darabi F, Nazari M, Tabatabaee HR (2013). Effects of Citizenship Education on Knowledge, Attitude, Subjective Norm and Behavioral Intention of High School Girls in Shiraz, Southern Iran. J Health Sci Surveillance Sys.

[32] Kupersmidt JB, Scull TM, Benson JW (2012). Improving Media Message Interpretation Processing Skills to Promote Healthy Decision Making About Substance Use: The Effects of the Middle School Media Ready Curriculum. J Health Commun.

[33] Page RM, Huong NT, Chi HK, Tien TQ (2011). Smoking media literacy in Vietnamese adolescents. J Sch Health.

[34] Kupersmidt JB, Scull TM, Austin EW (2010). Media literacy education for elementary school substance use prevention: study of media detective. Pediatrics.

[35] Whysall Z, Haslam C, Haslam R (2006). A stages of change approach to reducing occupational ill health. Prev Med.

[36] Callaghan RC, Herzog TA (2006). The relation between processes-of-change and stage-transition in smoking behavior: a two-year longitudinal test of the transtheoretical model. Addict Behav.

[37] Dino G, Kamal K, Horn K, Horn K, Kalsekar I, Fernandes A (2004). Stages of change and smoking cessation outcomes among adolescents. Addict Behav.

